# Prioritising Risk Factors for Type 2 Diabetes: Causal Inference through Genetic Approaches

**DOI:** 10.1007/s11892-018-1009-1

**Published:** 2018-05-19

**Authors:** Laura B. L. Wittemans, Luca A. Lotta, Claudia Langenberg

**Affiliations:** MRC Epidemiology Unit, University of Cambridge School of Clinical Medicine, Box 285 Institute of Metabolic Science, Cambridge Biomedical Campus, Cambridge, CB2 0QQ UK

**Keywords:** Genetics, Causal risk factors, Type 2 diabetes, Mendelian randomisation

## Abstract

**Purpose of the Review:**

Causality has been demonstrated for few of the many putative risk factors for type 2 diabetes (T2D) emerging from observational epidemiology. Genetic approaches are increasingly being used to infer causality, and in this review, we discuss how genetic discoveries have shaped our understanding of the causal role of factors associated with T2D.

**Recent Findings:**

Genetic discoveries have led to the identification of novel potential aetiological factors of T2D, including the protective role of peripheral fat storage capacity and specific metabolic pathways, such as the branched-chain amino acid breakdown. Consideration of specific genetic mechanisms contributing to overall lipid levels has suggested that distinct physiological processes influencing lipid levels may influence diabetes risk differentially. Genetic approaches have also been used to investigate the role of T2D and related metabolic traits as causal risk factors for other disease outcomes, such as cancer, but comprehensive studies are lacking.

**Summary:**

Genome-wide association studies of T2D and metabolic traits coupled with high-throughput molecular phenotyping and in-depth characterisation and follow-up of individual loci have provided better understanding of aetiological factors contributing to T2D.

## Introduction

### The Urgency to Prioritise Proposed Risk Factors

For very few of the many type 2 diabetes (T2D) risk factors [1–6, 7•] that have emerged from observational research has causal relevance been demonstrated [8–12]. Randomised controlled trials are the “gold-standard” for causality assessment but are expensive and time-consuming, and may not be feasible (e.g. birth weight, epigenetic markers) or ethical (e.g. alcohol consumption, smoking) for some exposures. Examples of high-profile failures to replicate interventions that were prioritised on the basis of observational evidence highlight the limited ability of even rigorously conducted observational epidemiological studies to control the influence of bias and confounding and allow inference about causality of the observed statistical associations [13, 14]. While risk factors that are not causally associated with a disease, for example markers of the subclinical disease process, can be useful for disease prediction, this is not the case if the aim is to identify targets for intervention. Hence, new ways to prioritise risk factors on the basis of their causal likelihood are needed.

### Genetic Approaches to Assess Causality

**Fig. 1:**
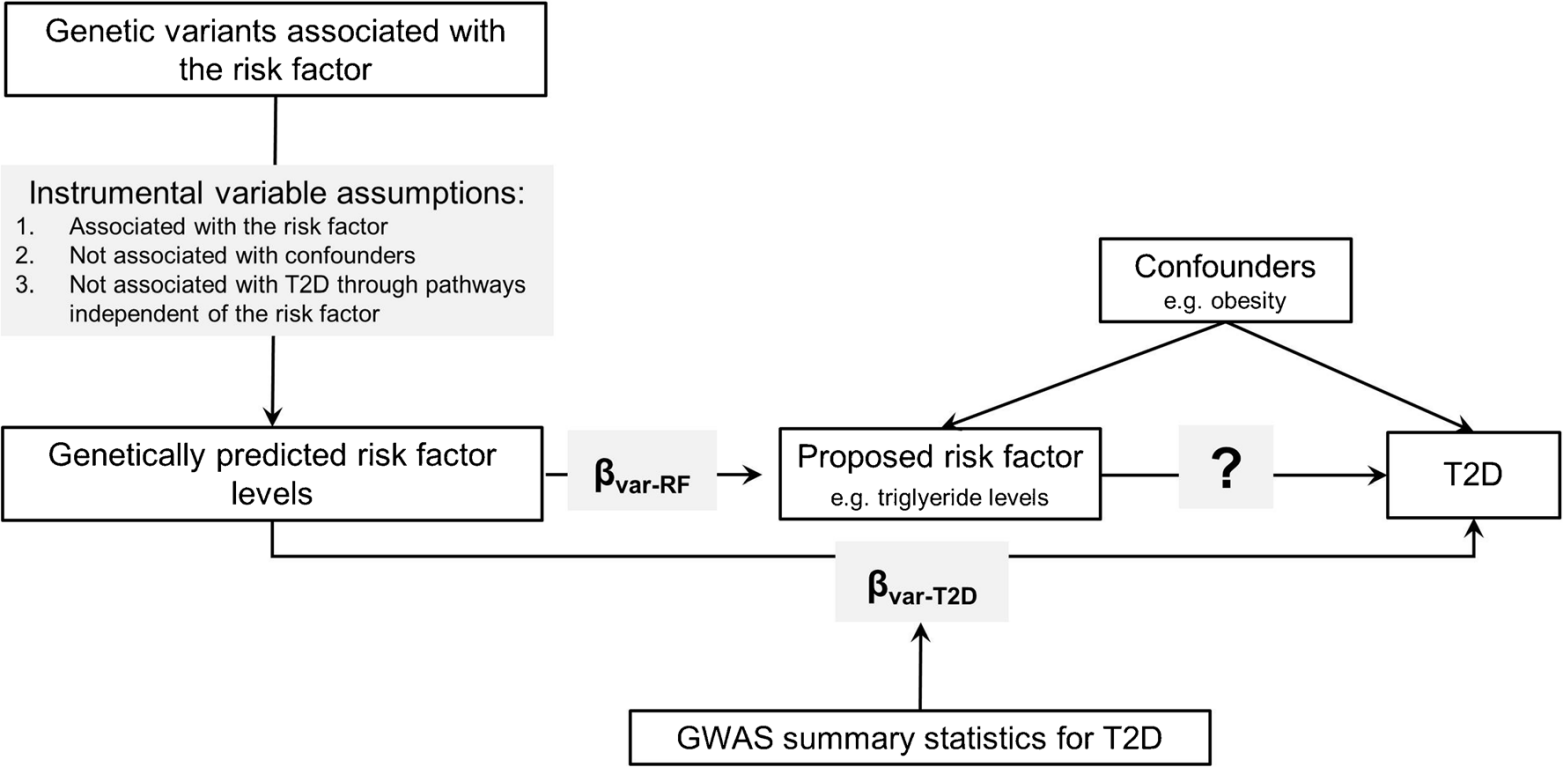
Schematic overview of the Mendelian randomisation (MR) framework. *β*_var-RF_: per-allele effect size of genetic variant on risk factor; *β*_var-T2D_: per-allele effect size of genetic variant on T2D

Genetic evidence through genotyping and sequencing of patients is expected to reduce the high rate of late-stage failures in drug development to translate laboratory models into the clinic [15–17]. Similarly, genetic insights may help to generate evidence about causal relevance and prioritise risk factors for testing in trials. Genetic approaches can support causal inference because genetic variants are generally less correlated with many of the measured and unmeasured factors that can confound observational exposure-to-disease associations, provided important assumptions are met (Fig. 1). This genetic approach to causal inference, referred to as “Mendelian randomisation”, was originally proposed for the investigation of the association between fibrinogen and coronary heart disease by Keavney [18] and has since gained popularity and been the subject of many investigations and reviews [19–22]. Genetic approaches to estimate causal effect sizes require estimates of genetic associations with exposures of interest and outcomes. For many risk factors, these data have already been generated through genome-wide association studies (GWAS), and results for T2D are publically available via the online type 2 diabetes knowledge portal (http://www.type2diabetesgenetics.org) [23]. Therefore, the cost and time investment is relatively small, specifically considering the costs, duration and participant burden of trials.

GWAS of T2D have clearly demonstrated the importance of established aetiological pathways, specifically pancreatic beta cell function, adiposity and insulin resistance [24••, 25, 26•]. This suggests that hypothesis-free genetic approaches provide a useful tool to identify and assess causality of novel disease mechanisms.

### Outline

This review focuses on aetiological pathways of T2D emerging from large-scale GWAS and richly phenotyped epidemiological studies, using three illustrative examples. We outline how recent genetic research has revealed a complex role of lipid metabolism in the aetiology of diabetes and has proposed peripheral fat storage capacity as a protective factor for T2D. We then describe how genetics can help to identify and prioritise causal pathways among the multitude of established and emerging biomarkers. Finally, we highlight the opportunities to use insights into the genomics of diabetes to infer its relevance as a causal risk factor for other common serious conditions, such as cancer.

## The Central Role of Peripheral Fat

### Overall Adiposity and Fat Distribution Both Affect Diabetes Risk

Obesity is a strong modifiable risk factor for T2D [8–12] and responsible for the secular increase in the diabetes prevalence, with three quarters of people with diabetes now living in low- and middle-income countries [27]. Weight loss interventions reduce diabetes risk [8–12] and are even able to revert glycaemia in patients with T2D to non-diabetic levels [28]. Genetic evidence supports the causal role of overall adiposity; the major body mass index (BMI) locus near *FTO* was the first found to exert its diabetes risk-raising effect entirely by increasing BMI. More recently, the largest European ancestry-focused GWAS of T2D, which tested 27 million variants and included more than 74,000 cases showed that 26 of the more than 400 conditionally independent variants at the identified loci for T2D predominantly increase risk through their effect on BMI, including established adiposity loci such as *FTO*, *MC4R* and *TMEM18* [24••]. Independent causal roles of overall adiposity and abdominal fat accumulation has also been supported by Mendelian randomisation studies [29•, 30, 31•].

### Genetic Loci Link Low Adiposity to High Diabetes Risk

Evidence is emerging that not all mechanisms leading to reduced fat accumulation are uniformly beneficial to metabolic health. Loci near *IRS1* and *COBLL1/GRB14* identified for lower total body fat percentage were found to be associated with higher risk of T2D [32•, 33]. Similarly, a coding variant in the *CREBRF* gene was found to increase BMI by 1.32–1.46 kg/m^2^ per allele but reduce diabetes risk by 40% in a Polynesian population [34•]. In line with this, the most recent T2D GWAS showed that associations for a subset of loci were strengthened following adjustment for body mass index; among these were signals previously shown to be associated with lower capacity of adipose tissue generation and expansion [35••], including *PPARG—*a gene involved in Mendelian forms of partial lipodystrophy [36]. Together, these results suggest that, when focussing on distinct pathways connecting adiposity and diabetes, certain biological processes may reduce fat accumulation while increasing risk of T2D, while others will reduce risk.

### Lipodystrophy-Like Mechanisms May Contribute to Insulin Resistance

Genetic research on metabolic risk factors demonstrated that distinct pathways linking low adiposity to diabetes risk may be driven by insulin resistance. It was recognised early on that discovery of loci associated with insulin resistance was facilitated by accounting for differences in obesity levels [37]; GWAS of insulin resistance have since been conducted based on fasting insulin levels adjusted for BMI [35••, 37–39]. Research on the relationship between insulin resistance and refined measures of body composition measured by dual energy X-ray absorptiometry (DEXA) showed associations between genetic determinants of insulin resistance and reduced fat accumulation in the legs and other peripheral compartments [35••, 39]. Positive relationships of insulin resistance with visceral fat mass and liver enzymes [35••, 39, 40] suggested that genetic predisposition to insulin resistance, not primarily driven by a long-term positive energy balance, is linked to a body fat distribution pattern that favours visceral and ectopic fat deposition over deposition in peripheral body compartments. Questions remain on whether impaired peripheral fat and enhanced central fat distribution are independent mechanisms.

Lotta et al. found that the 53 loci associated with hallmarks of common insulin resistance are also enriched in patients with familial partial lipodystrophy type 1, which is a rare condition characterised by extreme insulin resistance and the limited ability to store fat peripherally [35••]. These loci identified for common insulin resistance were enriched with causal genes involved in monogenic forms of lipodystrophy, including *PPARG*, *PIK3R1* and *INSR* [41]. This suggests the presence of shared mechanisms between common and rare forms of insulin resistance, and that lipodystrophy-like mechanisms may contribute to common forms of insulin resistance and metabolic and cardiovascular disease.

The majority of genetic studies have been conducted in participants of European ancestry. Many people who develop T2D in South-East Asia, the middle East and sub-Saharan Africa are not overweight or obese [42–44]. Ethnicity-specific patterns of fat distribution may underlie differences in metabolic risk for a given BMI, but the causal role of the relative contributions of greater abdominal and lack of gluteo-femoral fat have not been investigated.

### The Adipose Tissue Expandability Hypothesis

Altogether, these findings support the so-called adipose tissue expandability hypothesis, which states that each individual has a limited capacity to store excess energy in adipose tissue; once that threshold has been reached, excess energy in the form of lipids may accumulate ectopically in diabetes-relevant tissues such as the liver, muscles and the pancreas, where they may contribute to tissue dysfunction and diminished insulin action [45, 46]. Individuals with a larger capacity to peripherally accumulate fat may therefore be more protected from the cardio-metabolic consequences of a long-term positive energy balance. In support of this hypothesis, longitudinal data on weight change have demonstrated that people with a higher burden of insulin resistance-increasing alleles tend to expand their hip fat depot less as they gain weight than people at low genetic risk to develop insulin resistance [35••]. Refined imaging methods, including DEXA and magnetic resonance imaging (MRI), will help to further confirm this hypothesis and the specific contribution of these mechanisms to the broader relationship between fat distribution and metabolic risk in men and women and across ethnic groups.

## The Complex Interplay between Blood Lipid Fractions and T2D

### Genetics-Based Causality Assessments of Blood Lipids as Diabetes Risk Factors

After meta-analyses of randomised controlled trials of statins revealed that this major class of low-density lipoprotein cholesterol-lowering drugs modestly increases risk of T2D [47], a series of genetic studies focussing on the contribution of lipid fractions to diabetes risk was conducted. Early genetic studies investigating the causality of the blood lipid fractions focussed on the individual, separate contributions of three major lipid fractions: total triglycerides, high-density lipoprotein (HDL) and low-density lipoprotein (LDL) cholesterol [48–50]. Because levels of these fractions are regulated by partially overlapping physiological processes, genetic scores were not specific to one lipid fraction only, and therefore, specific causal inference is limited. To disentangle the causal effect of genetically related risk factors, the so-called multivariable Mendelian randomisation methods have been developed [51•], which estimate independent effect sizes of genetically correlated risk factors on the outcome in one model by taking the genetic correlation between them into account. High HDL cholesterol has been consistently associated with lower incidence of T2D, both observationally [52–54] and in multivariable Mendelian randomisation studies [55••, 56••], but recent evidence highlights that the extent to which LDL cholesterol and total triglyceride levels influence diabetes risk may be mechanism-dependent.

### Complex Relationships between LDL Cholesterol and Diabetes Risk

While there is no doubt that *higher* LDL cholesterol increases the risk of diseases of the heart and vasculature, strong evidence is now available that some mechanisms *lowering* LDL cholesterol increase the risk of T2D [55••, 56••]. Using multivariable Mendelian randomisation, two recent publications suggested that higher levels of LDL cholesterol are causally related to lower diabetes risk, in line with evidence of statin trials, [47] and a study of patients with familial hypercholesterolaemia, reporting a lower incidence of T2D, compared to their unaffected family members [57]. In contrast, familial hypercholesterolaemia was not associated with lower diabetes risk in an Amish population [58]. Differences in the genetic background, identification and management of these Dutch and Amish patient populations may have contributed to these discrepant findings.

The extent to which levels of LDL cholesterol influence diabetes risk may depend on the physiological mechanism that contributes to differences in lipid levels [59••, 60••]. For a given effect on LDL, the effect size of the associations between genetically predicted LDL cholesterol and T2D was shown to differ depending on the gene in which LDL variants are located when variants in current or prospective LDL-lowering drug targets, including *PCSK9*, *HMGCR*, *NPC1L1, ABCG5/G8* and *LDLR*, were compared [59••]. This is in contrast to the effect of LDL cholesterol on risk of coronary heart disease, which follows a clear log-linear pattern, in observational, genetic [59••, 61] and intervention studies [62].

### The Role of Total Triglycerides May Be Mechanism-Dependent

Although total triglycerides have been consistently associated with higher incidence of T2D [52–54], results for total triglycerides from genetics-based causal assessments are inconsistent [55••, 56••]. Genetic evidence suggests that triglycerides may be protective or detrimental for diabetes risk, depending on the physiological mechanism driving triglyceride levels. Triglyceride-lowering genes involved in intravascular lipolysis, including intravascular lipoprotein lipase (*LPL*) and its inhibitor angiopoietin-like 4 (*ANGPTL4*), have been associated with lower risk of T2D [35••, 60••], while triglyceride-lowering variants in genes involved in the hepatic production of triglyceride-rich lipoprotein particles were linked to higher risks of diabetes and fatty liver [60••]. In contrast, there is strong genetic evidence that several triglyceride-raising mechanisms are causally related to risk of coronary heart disease [60••, 63, 64].

These recent findings demonstrate how different genetic approaches can help to investigate and better understand the complex relationships between dyslipidaemia and diabetes risk by looking not only at risk factor levels, but specific mechanisms underlying these differences. Investigation of combined genetic scores that encompass a large range of biological mechanisms affecting overall lipid concentrations may help general inference about causality of the exposure in question but cannot reveal insight into the complexity underlying the regulation of lipid metabolism. Given that LDL loci (for a given effect on LDL) differed in their association with diabetes risk, locus-specific results may also help to better estimate the effect of interventions that target levels through different mechanisms will have on different outcomes. Pleiotropy and heterogeneity of genetic loci is considered a limitation of Mendelian randomisation, but studies on the role of lipid metabolism for diabetes aetiology highlight that biologically relevant insights can be obtained by investigating individual genetic loci that are pleiotropic or have, given their effect on the exposure, a disproportionate effect size on the outcome.

## Genetics to Prioritise Causal Pathways Amongst the Multitude of Reported Biomarker Associations

### Biomarkers as Causal Candidates

Interest into the potential causality of biomarkers for T2D has grown, as they can point to novel disease pathways and help to identify potential targets for intervention, provided they are causal. A systematic literature review has shown that more than 160 biomarkers measured in blood or urine have been proposed to be associated with T2D, but for a small minority has an assessment of causality been attempted [7•]. No evidence for causality was found for the majority of the biomarkers that have been investigated using genetic approaches, including Vitamin D [65], adiponectin [66, 67], uric acid [68], C-reactive protein [69] and gamma-glutamyl transferase [70–72]. However, for a few biomarkers, including sex hormone-binding globulin [73, 74], B and A-type natriuretic peptide systems [75, 76] and bilirubin [77], suggestive evidence for a causal relationship with T2D has been reported. Historically, some of the older studies investigating one biomarker at a time were based on comparatively small sample sizes and a limited understanding of the specificity of the genetic score. Replication of these findings is needed using genetic prediction models that maximise exposure variance explained, utilise largest available GWAS summary statistics, test generalisability across different ancestries where possible and investigate specificity of genetic variants in adequately powered studies with comprehensive coverage of relevant biological pathways.

### New Wave of Omics-Based Biomarkers

Technological developments in “-omics” methods have enabled high-throughput assessment of molecular traits in large epidemiological studies, including the metabolome, proteome and lipidome. This has led to a new wave and scale of observational diabetes “biomarker” discoveries, including metabolites [78–82], lipid species [83–86], proteins [87] and methylation markers [88•]. Molecular traits, such as metabolites, often represent the same biological pathway; therefore, large subsets of traits can be strongly correlated with each other. Cohort studies assessing different outcomes have shown that individual molecular traits tend to be associated with multiple disease outcomes [89–91]. The lack of specificity with regard to the exposure and outcome highlights that large-scale parallel biomarker assessment in an observational setting is subject to the same limitations as traditional biomarker studies and is even more prone to false-positive discovery by multiple testing. Integration of genetic data can be a helpful tool to prioritise potential causal pathways from the dense network of molecular trait-to-disease outcome associations.

### Omics Integration

GWAS have been conducted on the metabolome [92–94], proteome [95••] and methylome [96], with larger scale efforts underway. Results of these studies show that, due to the close biological proximity, genetic loci explain large proportions of the variance of a given marker and relevant biological mechanisms can be identified in relatively small sample sizes. For example, in a recent study on the genetic architecture of the human plasma proteome, GWAS were conducted for nearly 3000 proteins, based on 3000 participants [95••]. Despite the relatively modest sample size, more than 1900 genetic associations with 1478 proteins were found, and 66% of the identified regions influenced protein levels encoded by nearby genes. The median variance in protein levels explained by the variants was 5.8% (interquartile range 2.6–12.4%), which is similar the total variance in BMI explained by over 550 signals identified in a GWAS of around 700,000 participants [97].

Integration of genetic markers with large-scale molecular phenotyping can enable the identification of novel, molecular aetiological pathways. For example, GWAS in combination with metabolomics was recently used to assess the causality of the widely reported associations between branched-chain amino acids and incidence of T2D [89]. Based on a genetic score including variants in the *PPM1K* gene encoding the activator for the enzyme catalysing the rate-limiting step in branched-chain amino acid breakdown, evidence was found that branched-chain amino acid catabolism and T2D may be bidirectionally causally related [98•]. In another study, evidence based on cis-acting genetic markers was found that the protein marker macrophage metalloelastase 12 (MMP-12) may protect against coronary heart disease, while observational studies suggested a positive association [95••]. This finding suggests potential safety issues for MMP-12 inhibitors, which are being developed for the treatment of chronic obstructive pulmonary disease. Finally, Elliott et al. identified genetic loci associated with methylation markers previously associated with T2D and used these as genetic instruments to distinguish methylation markers that may be on the causal path to T2D from those that are likely to be non-causal [99••]. Their results suggested that the vast majority of methylation markers for diabetes are unlikely to be causal, with the exception of a methylation site at the *KCNQ1* locus [99••].

As -omics technologies are becoming more widely implemented in epidemiological studies of increasing size, opportunities to obtain an even more comprehensive insight into disease mechanisms may arise by integrating multiple layers of -omics data.

## The Causal Interplay between Diabetes, Metabolic Risk Factors and Cancer Incidence

Obesity [100] and T2D [101] have been linked to higher incidence of several cancers, but the causal role of metabolic health in cancer risk is difficult to assess observationally due to bias and confounding. Ongoing efforts by the Meta-Analyses of Glucose and Insulin-related traits Consortium (MAGIC) and others continue to improve our understanding of the genetic determinants of phenotypes associated with adiposity and T2D that are likely to contribute to the links between obesity, T2D and cancer. Application of these discoveries to results from GWAS identifying germline variants associated with site-specific cancer risk may help to refine our understanding of relevant pathways and identify appropriate preventative interventions. Such efforts critically depend on willingness and ability to make genome-wide summary statistics accessible for researchers in a timely manner. As mentioned above, the T2D research community systematically contributes new findings to an openly accessible online knowledge platform [23]. A similar dedicated online platform for cancer GWAS summary results and a more systematic deposition of GWAS summary results to existing online tools such as Phenoscanner (http://www.phenoscanner.medschl.cam.ac.uk/phenoscanner) [102] or MR Base (www.mrbase.org) [103] would be of great benefit to the scientific community and facilitate rapid and comprehensive investigation of the links between metabolic risk factors and cancer across different cancer sites and types.

Genetics-based research has proposed adiposity and insulin resistance as causal risk factors for specific types of cancer. Adiposity, insulin resistance and secretion have all been shown to be causally related to endometrial cancer risk, the site most strongly associated with obesity observationally [104, 105]. For breast cancer, a protective effect of body mass index in both pre- and post-menopausal breast cancer has been reported [106], but no causal role for T2D was found [107, 108]. Genetically predicted insulin resistance has also been associated with lung [109•] and pancreatic cancer [110], whereas genetically predicted BMI has been linked to higher risk of colorectal [111•], pancreatic [110] and lung cancer [109•].

These early genetics-based studies suggest that distinct metabolic risk factors may be causal for different types of cancer, but comprehensive investigations that assess the role of a range of refined metabolic risk factors across cancer types are still lacking.

## Conclusion

Rapid progress in the identification of the genetic basis of T2D and related phenotypes, together with technological advances that facilitate high-throughput and refined phenotyping at scale, provide important opportunities for epidemiological research to identify novel aetiological pathways and enhance causal understanding of potential risk factors that are amenable to intervention. Better powered studies now provide greater sensitivity to investigate pleiotropy that may have previously been overlooked and may have confounded effect sizes of genetically predicted exposures. Traditional genetic approaches to infer causality have generally focused on average effects, sometimes estimated from large polygenic scores. Recent examples have highlighted opportunities that arise from distinguishing the effects of specific mechanisms represented by a locus or a subset of loci, which may otherwise be overlooked.
